# Vertebral bone quality different in magnetic resonance imaging parameters

**DOI:** 10.1186/s13018-023-04268-5

**Published:** 2023-10-12

**Authors:** Xiang-Ge Liu, Xin Chen, Biao Chen, Pei-Jie Liang, Han-Hui Liu, Meiqi Fu

**Affiliations:** Department of Spinal Surgery, Foshan Fosun Chancheng Hospital, Fosun Group, Foshan, 528000 China

**Keywords:** Vertebral bone quality, VBQ, Spine, Osteoporotic vertebral compression, Fracture, Magnetic resonance imaging, Osteoporosis

## Abstract

**Objective:**

This was a single-center retrospective study that aimed to measure the vertebral bone quality (VBQ) in people of all ages and compare changes in VBQ across ages. Differences in VBQ under various MRI parameters were compared.

**Methods:**

We first screened patients without underlying disease and no history of fractures who underwent lumbar MRI in our center in the past four years. Over the span of 10 years, 200 patients (100 males and 100 females) were randomly recruited into each cohort to undergo 1.5 T and 3.0 T MRI scans. Subsequently, we tabulated the number of patients admitted to our hospital with OVCF over the past four years. There were 30 healthy adults under 4 times of MRI scans in different parameters to determine the differentiation of VBQ. The 30 healthy adults were recruited to validate the differentiation of VBQ under various parameters.

**Results:**

A total of 2400 patients without OVCF and 405 patients with OVCF were enrolled. The VBQ value of 1.5 T was significantly higher compared with that of 3.0 T (2.769 ± 0.494 > 2.199 ± 0.432, *P* < 0.0001). VBQ of 43.31 kHz in 1.5 T was significantly lower than that of 35.36 kHz (2.447 ± 0.350 < 2.632 ± 0.280, *P* < 0.05). The differentiation of VBQ in 1.5 T and 3.0 T was validated using results of healthy adults.

**Conclusions:**

VBQ is an effective tool for differentiating patients with OVCF and can be used as a primary screening tool for osteoporosis. However, VBQ is significantly affected by magnetic field intensity and bandwidth and cannot achieve its universality as it originally proposed.

## Introduction

Osteoporosis is a skeletal metabolic disease characterized by decreased bone mass and destruction of bone microstructure, which increases bone fragility and fracture risk. In 1994, BMD and T’s values were adopted as reference standards for osteoporosis, measured by dual-energy X-ray absorption (DXA) [1, 2]. Although current guidelines recommend an annual BMD test for women > 65 and men > 70 [3], less than 30% of eligible patients undergo one or more DXA tests [4].

Several methods are used to supplement DXA measurements, including quantitative computed tomography (q-CT), ultrasound bone density, and magnetic resonance imaging (MRI). Considering that older adults often undergo lumbar MRI for low back pain and lumbar disk herniation [5, 6], further rapid evaluation of osteoporosis by MRI may be effective methods for supplementing the currently used osteoporosis prevention systems [7].

It has been suggested that measuring bone marrow fat content on T1-weighted images can accurately reflect vertebral bone mass and predict compression fractures more effectively than DXA [8]. However, MRI machine and sequence parameters have a significant effect on the measurement of bone marrow fat, making it difficult to evaluate bone quality quantitatively in different regions, technicians, and machines. Therefore, the proposed score, VBQ [9], was determined using the T1-weighted ratio. This ratio is derived from the average signal intensity of the upper L1–L4 vertebral body in relation to the signal intensity of the cerebrospinal fluid (CSF) at the L3 level.

In our efforts to utilize VBQ for assessing patients’ bone quality, we discovered that the magnetic field intensity (MFI) has a substantial impact on VBQ measurements. This indicates that VBQ is less independent than initially suggested. To further explore this matter, we conducted this study to analyze the influence of various MRI parameters on VBQ.

## Methods and patients

Analysis of MRI images from 2400 patients does not infringe upon their rights. Therefore, the institutional review board waived the need for consent from the patients. However, 30 volunteers did provide informed consent to participate in the study, which was approval by the institutional review board of Foshan Fosun Chancheng Hospital (No. KY2022002). We enrolled patients who underwent lumbar MRIs at our center from April 2018 to April 2022. Exclusion criteria were as follows: (1) injection of MRI contrast material; (2) known oncologic patients; (3) traumatic vertebral injuries; (4) known or suspected demyelinating disease; and (5) presence of relevant image artifacts.

### VBQ in non-OVCF patients

Patients presenting with low back pain, lumbar disk herniation, or lumbar spinal stenosis were included. Patients with long-term corticospinal, parathyroid, diabetes, and fractures were excluded.

We restricted our patient count to those between the ages of 20 and 80. This decision was made due to the limited number of individuals over 80 who met the criteria. Additionally, there was a notable selection bias in our random screening process, largely due to the high prevalence of chronic diseases in the population. Therefore, we only counted up to the age of 80. A total of 200 patients (100 males and 100 females) in each age group who underwent 3.0 T and 1.5 T magnetic resonance scans of the lumbar spine were randomly selected at a 10-year stratification.

To evaluate the impact of MRI parameters on VBQ, we recruited 30 healthy adults aged 25 to 30 to receive multiple lumbar MRI using different parameters at our institution. The records of identified patients were screened to identify a history of congenital spine abnormalities, inborn bone metabolic disease, history of cancer, history of osteomyelitis, history of disseminated infection, history of chronic liver or kidney disease, or history of glucocorticoid use. Patients who met any of these criteria were not included in the healthy cohort.

### VBQ in OVCF patients

Patients with nonviolent and low-energy spinal compression fractures who had been followed up in our hospital for more than two years were included, and patients with vertebral fractures caused by severe violence and pathological fracture were excluded.

Demographic information, including age, sex, body mass index (BMI), and parameters of MRI, were collected.

### Parameters of MRI

The 1.5 T and 3.0 T magnetic resonance systems in this study were both from Siemens (Magnetom Sonata Maestro Class, Siemens Medical Solutions, Erlangen, Germany). The imaging protocol of 1.5 T sagittal T1-weighted spin-echo sequence: Repetition time (TR) = 645 ms; Echo time (TE) = 11 ms; slice thickness = 4 mm; squared field of view = 280 mm. Among 1200 patients, the bandwidth of more than ten patients was 35.36 kHz (158 patients), 36.06 kHz (100 patients), 37.03 kHz (120 patients), and 43.31 kHz (791 patients). Other frequencies used included 41.22 kHz (8 patients), 39.28 kHz (7 patients), 33.28 kHz (7 patients), 33.01 kHz (7 patients), and 32.2 kHz (2 patients).

The imaging protocol of 3.0 T sagittal T1-weighted spin-echo sequence: TR = 550 ms; slice thickness = 4 mm; and squared field of view = 280 mm. The combination of echo time and bandwidth frequency is: 56 kHz/9.4 ms (7 patients); 58 kHz/9.4 ms (9 patients); 64 kHz/9.4 ms (450 patients); 67.25 kHz/9.5 ms (10 patients); 69.5 kHz/9.5 ms (322 patients); and 76.75 kHz/9.5 ms (402 patients).

### Method of measuring VBQ

VBQ score was calculated using a non-contrast, T1-weighted MRI of the lumbar spine. First, midsagittal slices were used to measure the median signal intensity (SI) of the trabecular bone of the L1 through L4 vertebral bodies. Next, for patients with abnormalities that prevented region-of-interest (ROI) measurement on the midsagittal slice (e.g., hemangioma, venous plexus, scoliotic changes, fracture, bone marrow edema), parasagittal slices were used to have SIs accurately reflect inner medullary portions of bone. Finally, in cases where abnormalities invaded all sagittal slices of the vertebral body, this level was excluded from the calculation, and the VBQ was determined using only the remaining vertebrae.

**Fig. 1:**
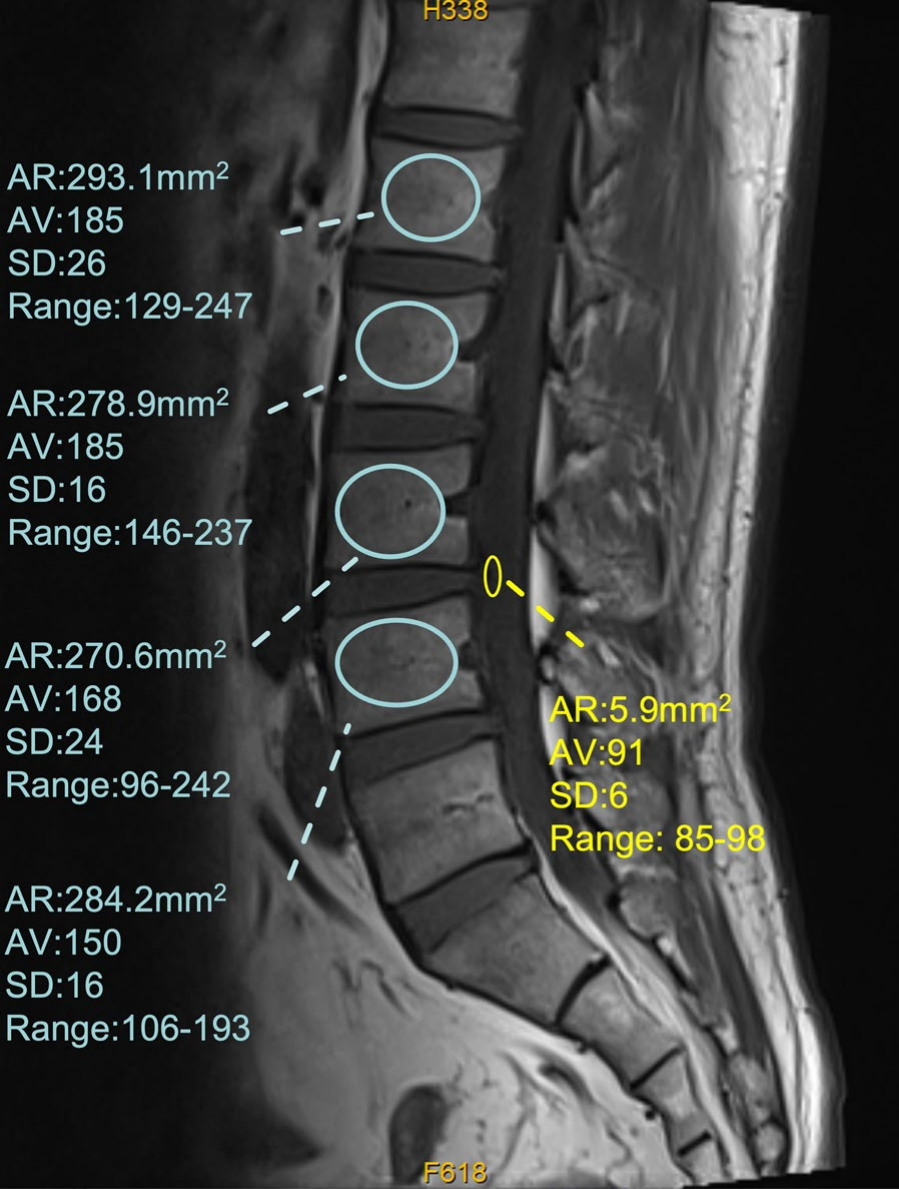
Non−contrast-enhanced T1-weighted MRI of the lumbar spine detailing the regions of interest (circles) used to calculate VBQ score; AR= area ratio; AV = average signal intensity of the region of interest; SD= standard deviation of signal intensity of the region-of-interest (VBQ score in shown example=2.291)

The median value of L1–L4 vertebral bodies is then determined and divided by the SI of the adjacent CSF to provide a relative vertebral body SI: the VBQ (Fig. 1). The SI of CSF was pegged to the median SI within an ROI placed at the level of L3 to allow for standardization of this step. In cases where L3 CSF space was obstructed entirely by descending nerve roots, CSF ROI was placed at the level of L2 or L4.

$$VBQ = \frac{{SI_{L1 - 4} }}{{SI_{CSF} }}$$

While calculating VBQ, we placed no restrictions on MRI field strength or scanner manufacturer. All VBQ measurements were made by two independent reviewers blinded to clinical outcomes. VBQ values used in the statistical analysis represent the average of the values calculated by the two reviewers. A third reviewer was used in cases where the scores differed by more than 10%, and the value used for analysis was the average of the two closest VBQ measurements.

### Statistical analysis

All data were collected using Microsoft Excel (Redmond, WA, USA) and analyzed with Prism version 9.0 for Mac (GraphPad Software Inc., San Diego, California, USA). Descriptive statistics constituted mean and standard deviation for continuous variables and proportions for dichotomous and categorical variables. The univariable analysis included a two-tailed Student t test for continuous variables and a chi-square analysis for categorical variables. The multivariable model was analyzed using logistic regression and included all clinically relevant variables.

## Results

### Patient characteristics

A total of 2,400 non-OVCF patients were included in the analysis. The mean age was 50.21 ± 16.32 years, and the mean BMI was 23.78 ± 3.60 kg/m^2^. Of 405 vertebral compression fractures, 330 were females (age 75.06 ± 8.35; BMI 22.16 ± 3.79 kg/m^2^), and 75 were males (age 79.16 ± 8.35; BMI 21.01 ± 3.13 kg/m^2^). The mean age of the healthy adults was 27.3 ± 1.7 years, and the mean BMI was 24.1 ± 3.5 kg/m^2^ (Table 1).

**Table 1:** Demographics and VBQ score of 30 healthy adults

Variable	Mean
Age	27.3 ± 1.7
Female	15(50%)
Height (cm)	160.1 ± 9.5
Weight (kg)	61.9 ± 11.6
BMI (kg/m^2^)	24.1 ± 3.5

*VBQ* vertebral bone quality

### VBQ in different MFI, TE, and Bandwidth

**Fig. 2:**
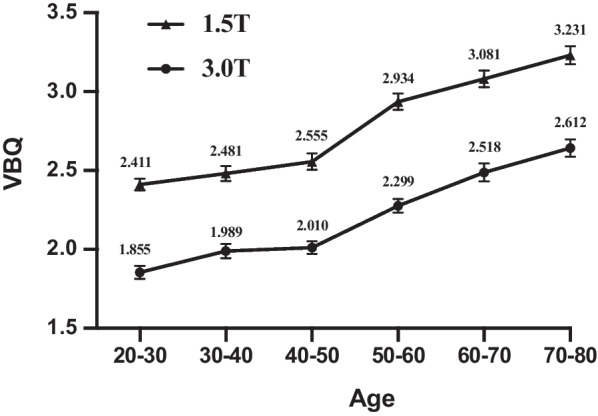
VBQ changes with age at different magnetic-field strengths

**Fig. 3:**
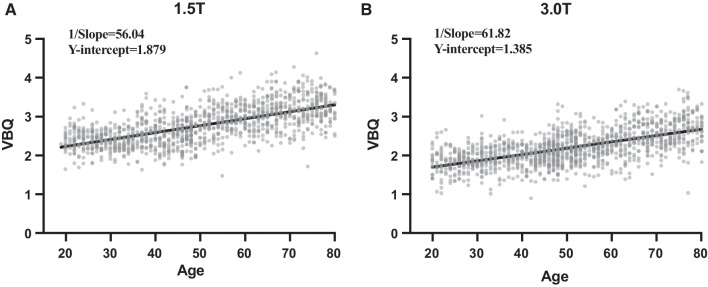
Simple regression curve of VBQ with age at different field intensities

VBQ was 2.769 ± 0.494 at 1.5 T and 2.199 ± 0.432 at 3.0 T (*P* < 0.0001). There was also a significant difference in VBQ between 1.5 T and 3.0 T in all age groups (Fig. 2). *R*^2^ of simple regression analysis between VBQ and age was 0.4144 (*P* < 0.0001) in 1.5 T and 0.3647 (*P* < 0.0001) in 3.0 T (Fig. 3). There was no statistical difference in slope between the two regression curves.

To mitigate the age-related variations, we focused our analysis on individuals aged 30 to 50 years with more than ten cases, considering the combination of field intensity, TE, and bandwidth. In 1.5 T, VBQ bandwidth of 35.36 kHz, 36.06 kHz, 37.03 kHz, and 43.31 kHz was compared between the two groups, and there was a statistical difference between 35.36 and 43.31 kHz. In 3.0 T, there was no statistical difference between TE 9.4 ms/bandwidth 64 kHz, TE 9.5 ms/bandwidth 69.5 kHz, and TE9.5 ms/bandwidth 76.75 kHz (Table 2).

**Table 2:** Different VBQ in different magnetic field strength, echo time, and bandwidth

Magnetic field intensity	Echo time (ms)	Bandwidth (kHz)	Age	Sex (Female)	VBQ	*P* value
1.5 T	11	35.36	41.5 ± 10.7	24 (36/66.7%)	2.632 ± 0.280	*P* = 0.0342*
	11	36.06	40.5 ± 9.2	14 (44/31.8%)	2.551 ± 0.395	*P* > 0.05
	11	37.03	40.4 ± 6.1	46 (106/43.4%)	2.544 ± 0.402	P > 0.05
	11	43.31	40.2 ± 8.3	101 (238/46.2%)	2.447 ± 0.350	*P* = 0.0241*
3.0 T	9.4	64	42.7 ± 7.8	112 (202/55.4%)	2.074 ± 0.328	P > 0.05
	9.5	69.5	41.8 ± 7.4	96 (176/54.5%)	2.019 ± 0.388	*P* > 0.05
	9.5	76.75	41.9 ± 7.7	76 (158/48.1%)	2.014 ± 0.305	*P* > 0.05

*VBQ* vertebral bone quality

^*^Post hoc analysis revealed that the VBQ in 1.5 T/11 ms/35.36 kHz group was significantly lower than that in 1.5 T/11 ms/43.31 kHz group

### VBQ in different age and sex

**Fig. 4:**
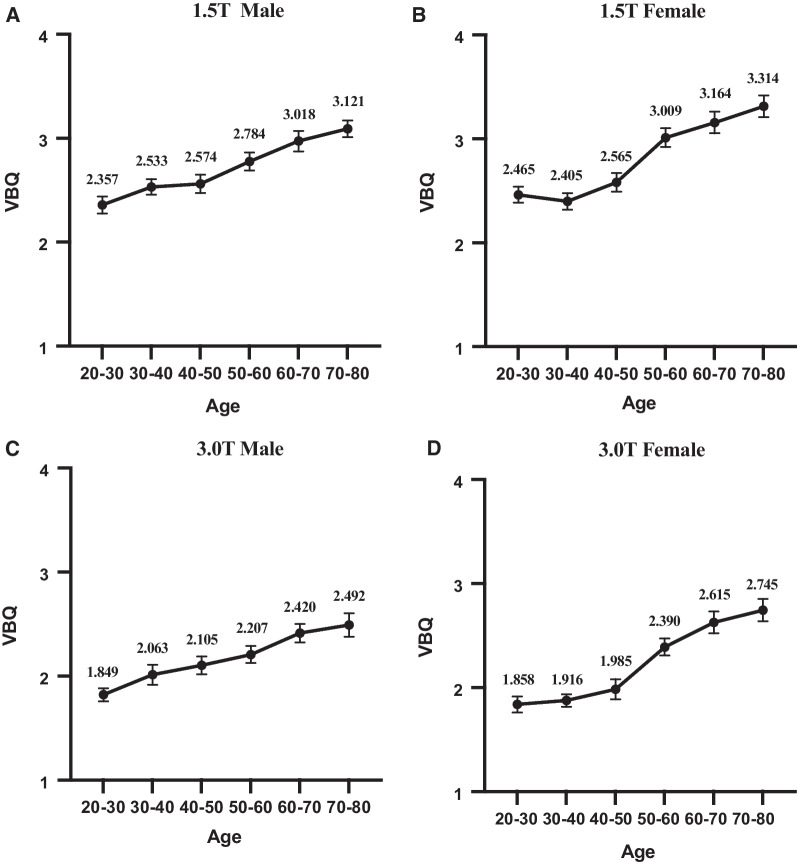
VBQ changes with age at different Magnetic-field strengths and different sex

VBQ of both men and women increased with age, but the pattern of increase was different (Fig. 4). VBQ of males increased gradually. For example, in 1.5 T, VBQ increased from 2.357 ± 0.371 to 3.121 ± 0.390, an increase of 32.41%; in 3.0 T, it increased from 1.849 ± 0.217 to 2.492 ± 0.438, an increase of 34.78%.

Female VBQ ages 20–40 decreased from 2.465 ± 0.290 to 2.405 ± 0.301 in the 1.5 T group. However, there was a significant increase between the ages of 40 and 60. The 1.5 T group increased from 2.565 ± 0.361 to 3.009 ± 0.365, with an increase of 16.90%. The 3.0 T group increased from 1.985 ± 0.314 to 2.390 ± 0.268, with an increase of 20.40%. In general, female VBQ in the 1.5 T group increased from 2.462 ± 0.290 to 3.314 ± 0.402, 34.65%. The 3.0 T group increased from 1.838 ± 0.227 to 2.745 ± 0.400, 49.35%.

### VBQ in OVCF patients

**Fig. 5:**
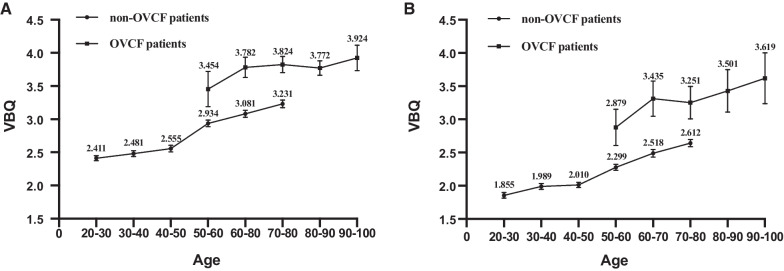
Comparison between patients with OVCF and without OVCF in different magnetic field intensity. A. Comparison in 1.5T; B. Comparison in 3.0T

The VBQ value of OVCF patients was 3.775 ± 0.590 in 1.5 T, which was 3.692 ± 0.510 in males and 3.794 ± 0.606 in females. It was 3.285 ± 0.878 in 3.0 T, with 3.017 ± 0.626 for males and 3.342 ± 0.628 for females. VBQ was significantly higher in patients with OVCF than in non-OVCF patients (Fig. 5).

VBQ values were 3.309 ± 0.995 and 3.292 ± 0.769 (*P* > 0.05) at 3.0 T in patients with past and first compressive fractures, respectively. In 1.5 T, they were 3.687 ± 0.572 and 3.879 ± 0.594, respectively (*P* < 0.05).

### VBQ in healthy adults

**Fig. 6:**
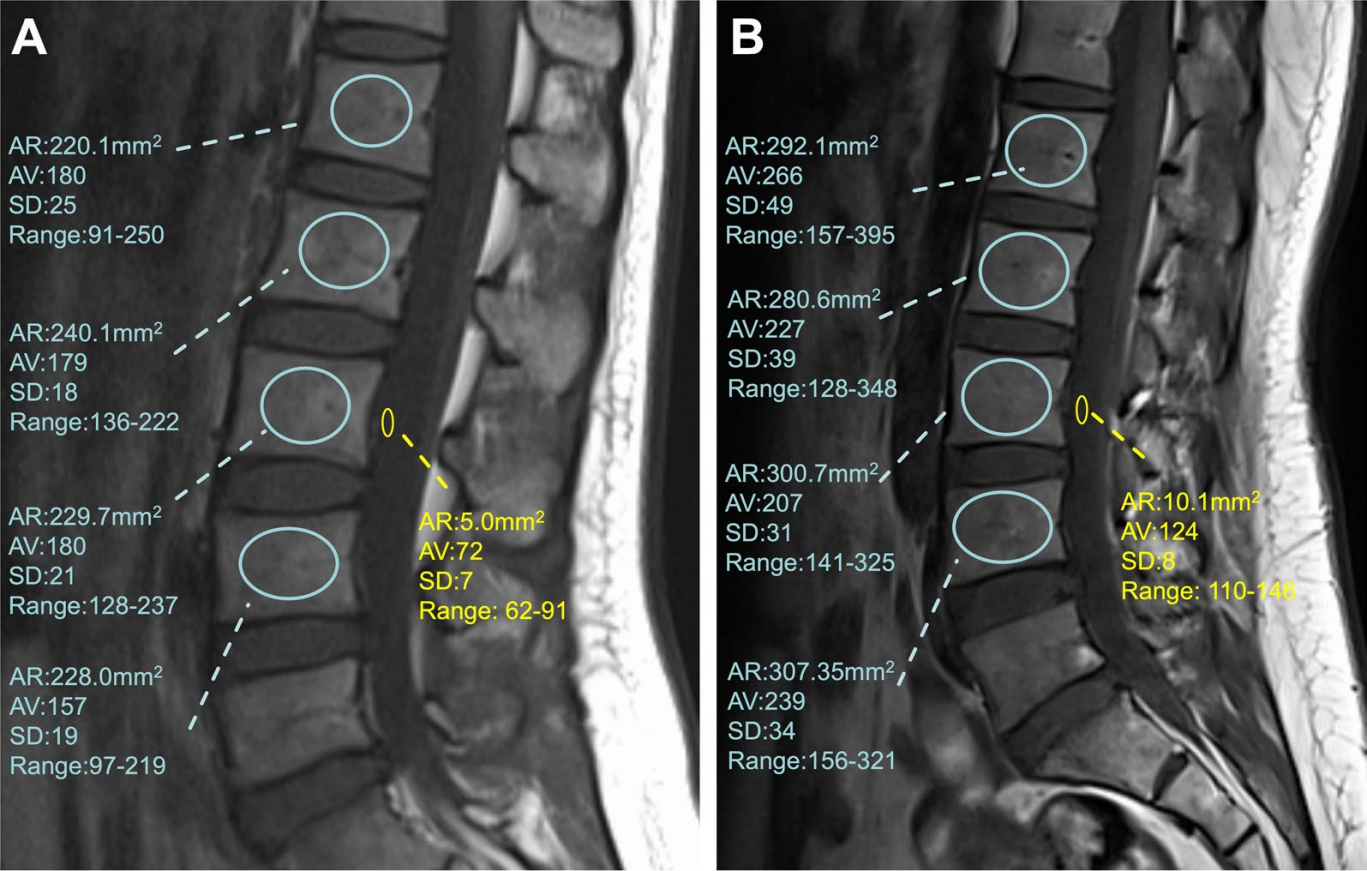
Different VBQ of one 27 years-old-volunteer in 1.5T and 3.0T.(A. VBQ in 1.5T is 2.417; B. VBQ in 3.0T is 1.893)

We recruited 30 healthy adults, who underwent multiple MRI scans, to validate the above retrospective results. The mean VBQ value was 2.398 ± 0.281 at a magnetic field intensity of 1.5 T, and 1.817 ± 0.229 at a magnetic field intensity of 3.0 T (Fig. 6). At different bandwidths of the same magnetic field intensity, the larger bandwidth had a smaller VBQ (Table 3).

**Table 3:** Different VBQ of healthy adults in different magnetic field strength, echo time, and bandwidth

Variable			VBQ	*P*
Magnetic field intensity (T)	Echo time (ms)	Bandwidth (kHz)		
1.5	11	37.03	2.419 ± 0.312	0.173
	11	43.31	2.367 ± 0.281	
3.0	9.5	69.5	1.831 ± 0.221	0.528
	9.5	76.75	1.816 ± 0.254	

*VBQ* vertebral bone quality

## Discussion

Although DXA is currently the gold standard for diagnosing bone loss, Dipaola et al. showed that only 44% of patients underwent dual-energy X-ray bone mineral density examination before internal lumbar fixation and fusion surgery [10]. In the absence of BMD results, spinal surgeons face a challenge in evaluating patients for osteoporosis. In addition, patients undergoing surgery for lumbar diseases often receive routine lumbar MRI, so some doctors try to assess osteoporosis status in patients with lumbar MRI [8, 9, 11, 12].

Over the past, researchers mainly explored strategies for MR fat quantification. Bone marrow adipose tissue dilatation is considered a space-filling process that occurs after bone mass decreases [13]. Bone marrow fat (BMF) was negatively correlated with BMD in patients with osteoporosis [14, 15]. However, BMF was higher in patients with brittle fractures after adjustment for age, sex, and BMD [16, 17]. Among them, the increase in BMF was strongly related to vertebral compression fracture [18]. Bandirali et al. found that the accuracy of bone mass fraction (M value) based on BMF in MRI predicting osteoporosis was 84%. The sensitivity of the M value in predicting osteoporosis was higher than that of the T value and was not affected by degenerative osteoarthritis and obesity [8]. However, M values varies significantly under different magnetic resonance systems, which is a major limitation.

To eliminate the influence of different magnetic resonance systems, Ehresman et al. introduced the parameter of CSF signal intensity while utilizing MRI images to evaluate patients’ BMD before lumbar fusion internal fixation [9].

To date, studies have suggested that a higher VBQ score correlate with screw loosening and reoperation after internal lumbar fixation [19, 20]. Furthermore, the ability to predict new vertebral compression fractures using the VBQ score in combination with baseline bone mass can be achieved [11].

The aim is to eliminate individual differences without interference from inspection instruments so that measurements can be made between different types of inspection instruments [21]. However, we found that VBQ value is not as independent of the machine’s inspection instrument as the proposer thought.

First, the MFI has a significant influence on VBQ. The VBQ value of 3.0 T is less than that of 1.5 T. In MRI, the value of T1 depends on the thermal transition probability. The greater the transition energy, the greater the probability of the transition. The transition energy of the 3.0 T system is larger than that of the 1.0 T and 1.5 T systems. Therefore, the energy transfer capacity of H protons in tissues decreases with the increase in the external magnetic field, and the time for the longitudinal magnetization vector to recover to the maximum value of 63% is longer. As a result, the T1 relaxation time of 3.0 T devices is 1.1–1.4 times longer than that of 1.5 T devices during MRI.

This is an even more significant difference in the spin-echo sequence. Because the traditional spin echo is usually stimulated by a constant 90° or 180° pulse, the echo signal is collected, and a phase coding line is filled in the K space. When the excitation angle is 90° or 180°, T1 component is the largest, and T1 contrast is the strongest. However, since the excitation angle is 90° or 180°, longitudinal relaxation must be relaxed from 0 or the inverse maximum magnetization vector, thus the imaging time is very long. T1 of 3.0TMR has lower contrast than 1.5 T under the same conditions.

Bandwidth also has a certain impact on VBQ. As there is no study to find the influence of bandwidth on VBQ value, we only put forward a hypothesis. This phenomenon may cause by false excitation in chemical shift imaging. The resonance frequency of fat is about 3.4 ppm lower than that of water. During radio frequency excitation, radio frequency pulses with a certain bandwidth need to be used to stimulate tissues. However, the resonance frequencies of fat and water from the same physical location are different, leading to false excitation of the spatial location of water fat, and the fat signal in the direction of higher frequency of the selection layer is excited. In the process of frequency coding, the fat signal is mismatched to the lower frequency coding direction due to the difference in the precession frequency of water fat. These causes chemical shift artifact between water and fat decreases with increasing bandwidth and increases with decreasing bandwidth. In 1.5 T, although there is only a statistical difference between the female group at 33.01 kHz and 43.31 kHz, VBQ value decreases gradually with the decrease of bandwidth. In 3.0 T, VBQ value of 69.5 kHz was also larger than that of 76.75 kHz, although there was no statistical difference.

### Limitation

First, given the retrospective design, of this study the time span of MR images in our study is long, resulting in large differences between MR image parameters. While this also allows us to compare differences between different parameters, it reduces the credibility of VBQ values. Second, we could not directly compare the effectiveness of BMD and VBQ because few eligible patients in our center underwent DXA bone mineral density examination. Finally, we compared the differentiation of VBQ between non-OVCF patients > 50 years old and OVCF patients, but this was not a comprehensive cohort comparison. In this study, the average age of non-OVCF patients > 50 years old was 65.32 ± 8.92 years old, much smaller than that of patients with OVCF. The bias caused by age and underlying diseases cannot be ignored.

## Conclusion

VBQ attempts to balance differences between machines and operators by the strength of CSF signals. However, our preliminary study shows that the VBQ value is significantly differs under various field strengths, and even a certain difference exists between different bandwidths in the same field strengths. Currently, MR can only be used as a preliminary screening for osteoporosis as it is greatly affected by various parameters described in this paper.
